# Human plasma-derived eNAMPT-containing extracellular vesicles promote NAD^+^ biosynthesis and thermogenesis in mice

**DOI:** 10.1038/s41514-025-00297-y

**Published:** 2025-11-24

**Authors:** Kiyoshi Yoshioka, Takumi Sugimoto, Mamoru Oyabu, Naoki Ito, Aoi Kodama, Yasutomi Kamei, Shin-ichiro Imai

**Affiliations:** 1 Institute for Research on Productive Aging (IRPA), Tokyo, Japan; 2 Brain-Skeletal Muscle Connection in Aging Project Team, Geroscience Research Center, National Center for Geriatrics and Gerontology, Obu, Japan; 3 Graduate School of Life and Environmental Sciences, Kyoto Prefectural University, Kyoto, Japan; 4 Department of Developmental Biology, Department of Medicine, Washington University School of Medicine, St. Louis, MO USA

**Keywords:** Ageing, Therapeutics

## Abstract

Age-associated decline in tissue NAD^+^ levels contribute to functional impairments, recognized as aging. Nicotinamide phosphoribosyltransferase (NAMPT), the rate-limiting enzyme crucial for NAD^+^ biosynthesis in mammals, is encapsulated in extracellular vesicles (EVs) and secreted into the bloodstream. The importance of extracellular NAMPT-containing EVs (eNAMPT-EVs) in hypothalamic NAD^+^ biosynthesis has been demonstrated in several mouse models. However, whether eNAMPT-EVs derived from human plasma can also act as a physiological NAD^+^ booster remains unclear. Here we show that administration of human plasma-derived, highly purified eNAMPT-EVs can elevate hypothalamic NAD^+^ levels in mice. Furthermore, eNAMPT-EV administration led to an increase in body temperature and suppression of hypothalamic *Npy* gene expression. These responses were negated by pharmacological NAMPT inhibition. We also found that exercise increases in plasma eNAMPT and hypothalamic NAD^+^ levels. These findings suggest that enhancing circulating eNAMPT-EVs can be an effective strategy for NAD^+^ boosting and potentially an effective anti-aging intervention in humans.

## Introduction

Nicotinamide adenine dinucleotide (NAD^+^) is an essential coenzyme in a myriad of redox reactions and also a substrate for NAD^+^-consuming enzymes, such as sirtuins, poly-ADP-ribose polymerases (PARPs), CD38/157 NAD^+^ hydrolases, and sterile alpha and Toll/interleukin-1 receptor motif-containing 1 (SARM1). Through these reactions, NAD^+^ plays a pivotal role in the regulation of many fundamental cellular processes, including metabolism, autophagy, DNA repair, epigenetics, stress response, inflammation, and aging^1–5^. Over the past decade, it has been demonstrated that the levels of NAD^+^ decrease in various tissues over age, causing age-related functional deterioration and diseases. Therefore, “NAD^+^ boosting,” which aims to replenish NAD^+^ to prevent and ameliorate age-associated functional decline, has been gaining a significant attention as an effective anti-aging intervention^6–9^.

Nicotinamide phosphoribosyltransferase (NAMPT) is the rate-limiting enzyme for NAD^+^ biosynthesis in mammals and plays a critical role in a variety of tissue functions and disease pathogenesis^10–12^. In particular, it has been shown that adipose tissue actively secretes extracellular NAMPT (eNAMPT) into blood circulation by encapsulating it into extracellular vesicles (eNAMPT-EVs)^13,14^. This process requires the NAD^+^-dependent deacetylation of lysine 53 on intracellular NAMPT (iNAMPT) by SIRT1, a main member of mammalian sirtuins^15^. Circulating eNAMPT-EVs are distributed to multiple tissues, including the hypothalamus, a central hub for aging and longevity control^16–18^. eNAMPT is internalized into the cytoplasm, immediately promoting NMN/NAD^+^ biosynthesis in target tissues. We have previously demonstrated that administration of eNAMPT-EVs collected from the plasma of young mice leads to significant increases in NAD^+^ in the hypothalami of aged mice, delaying aging and extending maximal lifespan in mice^13,19^. These findings have implicated a potential application of eNAMPT-EVs as a novel anti-aging NAD^+^ booster.

In this study, using ultracentrifuge-purified human plasma-derived eNAMPT-EVs, we investigated whether human eNAMPT-EVs could be used for NAD^+^ boosting in cells and mice. We also examined the physiological effect of voluntary exercise on circulating eNAMPT-EVs and found that circulating eNAMPT levels were significantly increased by a single bout of treadmill exercise, boosting NAD^+^ levels in the hypothalami of aged mice subjected to long-term voluntary running exercise. Furthermore, we observed increased rectal body temperature and gene expression changes responsible for thermoregulation in the hypothalamus when supplementing with human eNAMPT-EVs in mice. Taken together, our findings demonstrate that the NAD^+^-boosting function of eNAMPT-EVs is conserved between mice and humans, suggesting the potential of human plasma-derived, highly purified eNAMPT-EVs as an effective agent for NAD^+^ boosting in humans.

## Results

### Mouse plasma eNAMPT is internalized and promotes NAD^+^ biosynthesis in HEK293 cells and mice

**Fig. 1:**
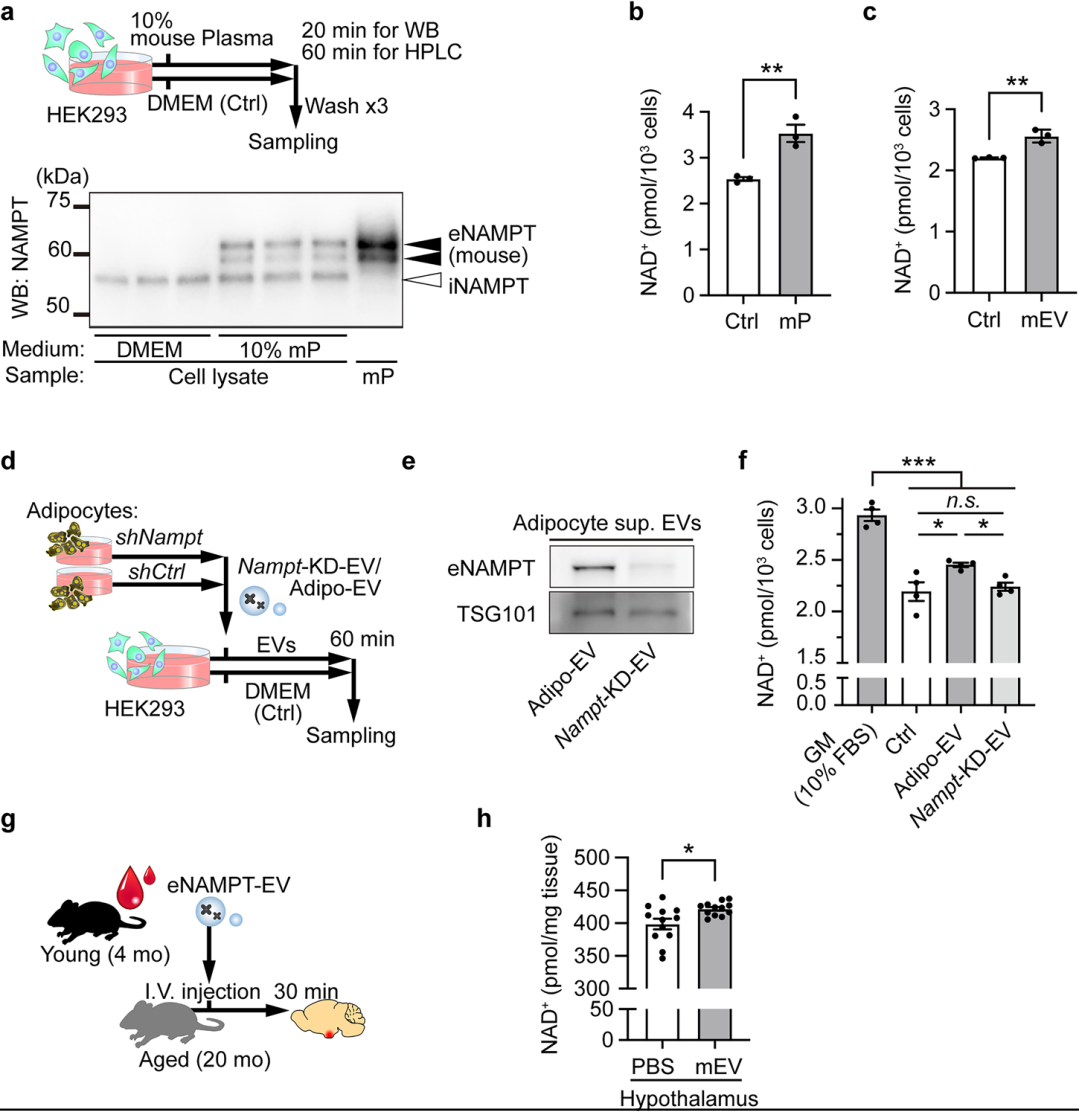
Mouse plasma and its eNAMPT-EVs increase NAD^+^ in cultured cells and the hypothalamus. **a** A scheme showing the experimental protocol and immunoblot results showing eNAMPT uptake in HEK293 cells upon addition of 10% mouse plasma (mP). The lane labeled mP shows eNAMPT in mouse plasma. Cells treated with DMEM containing 1% penicillin but no FBS were used as a control (Ctrl). **b**, **c** Increases in NAD^+^ in recipient HEK293 cells upon addition of 10% mouse plasma (**b**) and extracellular vesicles (mEV) purified by ultracentrifugation from an equivalent volume of 10% mouse plasma (**c**) (*n* = 3). **d** The scheme to examine the effect of eNAMPT-EVs released from control and *Nampt*-KD OP9 adipocytes on cellular NAD^+^ levels in recipient HEK293 cells. Cells treated with DMEM containing 1% penicillin but no FBS were used as a control (Ctrl). **e** Western blot analysis of eNAMPT contained in EVs collected from control and *Nampt*-KD OP9 adipocyte culture supernatants via ultracentrifugation (Adipo-EV vs. *Nampt*-KD-EV). **f** NAD^+^ levels in recipient HEK293 cells incubated with EVs derived from control and *Nampt*-KD OP9 adipocytes (*n* = 4). **g** A scheme of eNAMPT-EV administration in mice. **h** Hypothalamic NAD^+^ levels following intravenous administration of mouse plasma-derived eNAMPT-EVs (*n* = 12). One, two, and three asterisks indicate *p* < 0.05, *p* < 0.01, and *p* < 0.001, respectively. Unpaired Student’s t-test was conducted to compare the results shown in (**b**), (**c**), and (**h**), and one-way ANOVA with Holm-Sidak’s multiple comparison test was used for (**f**). Data are presented as mean ± SEM.

We first aimed to establish a bioassay in which the NAD^+^ biosynthetic activity of eNAMPT-EVs could be measured. For this purpose, we employed a human embryonic kidney cell line HEK293, a common cell line that has been used in the biomedical research field. When incubating HEK293 cells with 10% mouse plasma for 20 min, we were able to clearly confirm the internalization of mouse eNAMPT, which is often detected as dual bands of higher molecular weights than iNAMPT in Western blot analyses (Fig. 1a). In this condition, we were also able to detect significant increases in intracellular NAD^+^ levels in HEK293 cells at 60 min time point after adding 10% mouse plasma (Fig. 1b). We then used EVs purified from an equivalent volume of 10% mouse plasma by ultracentrifugation. Ultracentrifuge-purified eNAMPT-EVs successfully increased NAD^+^ levels in HEK293 cells (Fig. 1c). In our previous study, we have demonstrated that whereas EVs purified from the culture media of OP9 adipocytes significantly enhance NMN biosynthesis in primary hypothalamic neurons, EVs purified from the culture media of *Nampt*-knockdown (*Nampt*-KD) OP9 adipocytes fail to enhance NMN biosynthesis^13^. Therefore, we also examined whether the observed NAD^+^ increase in HEK293 cells was also due to eNAMPT contained in EVs. Control OP9 adipocytes significantly increased both mRNA and protein expression levels of NAMPT at day 4 of their differentiation (Supplementary Fig. 1a and b). Because newly generated *Nampt*-KD OP9 cells were able to differentiate into adipocytes properly (Supplementary Fig. 1b and c), we collected EVs from the culture supernatant of *Nampt*-KD OP9 adipocytes, purified them by ultracentrifugation, and administered to HEK293 cells (Fig. 1d). EVs from *Nampt*-KD OP9 adipocytes contained very faint levels of eNAMPT and failed to elevate NAD^+^ levels in HEK293 cells (Fig. 1e and f), clearly demonstrating that eNAMPT contained in EVs is responsible for NAD^+^ increase in recipient cells. We also confirmed the NAD^+^ biosynthetic activity of purified eNAMPT-EVs in vivo. When eNAMPT-EVs purified from plasma of 4 month-old mice were administered to 20 month-old mice, hypothalamic NAD^+^ levels were significantly increased (Fig. 1g and h). Consistent with our previous results, these results clearly indicate that eNAMPT-EVs purified from mouse plasma exhibit NAD^+^ biosynthetic activity in both cultured cells and mice.

### Human plasma-derived, ultracentrifuge-purified eNAMPT-EVs promote NAD^+^ biosynthesis in cultured cells and mice

**Fig. 2:**
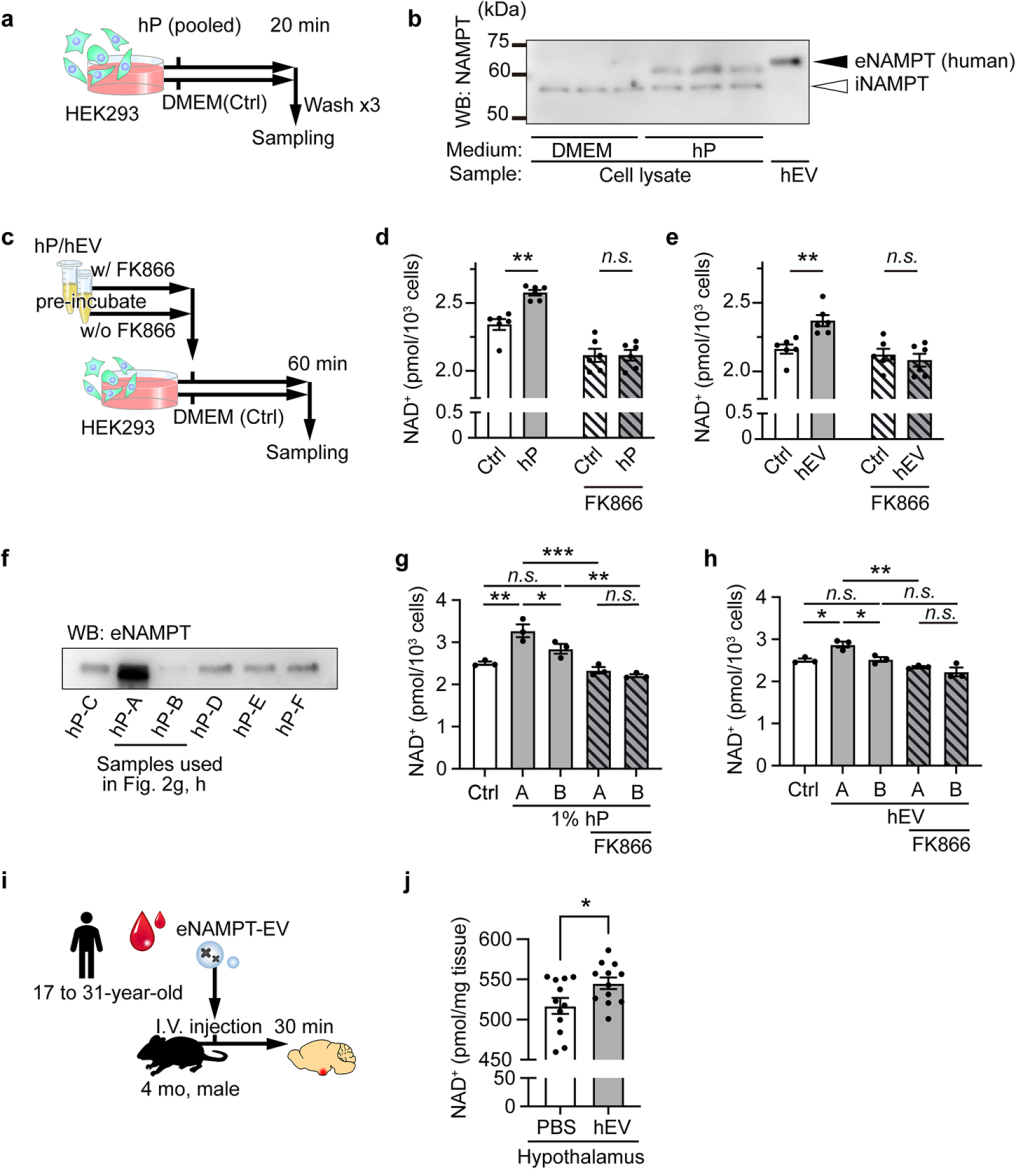
eNAMPT-EVs purified from human plasma enhance NAD^+^ biosynthesis in cultured cells and mice. **a** A scheme illustrating the protocol for human eNAMPT uptake in HEK293 cells. Cells treated with DMEM containing 1% penicillin but no FBS were used as a control (Ctrl). **b** Immunoblot results showing eNAMPT uptake in recipient HEK293 cells upon addition of 10% human plasma (hP). The lane labeled hEV shows eNAMPT contained in EVs purified from human plasma. **c** A scheme illustrating the protocol for addition of human plasma (hP) or human plasma-derived EVs (hEV), with or without pre-incubation with FK866 (final concentration, 100 nM, for 3 hours). Cells treated with DMEM containing 1% penicillin but no FBS were used as a control (Ctrl). **d**, **e** NAD^+^ levels in recipient HEK293 cells (*n* = 6). 2% hP or hEV collected from a 5%-equivalent volume of plasma was added. Two-way ANOVA revealed a significant interaction between hP/hEV and FK866 (hP × FK866, *p* < 0.01; hEV × FK866, *p* < 0.05). **f** Western blot analysis of eNAMPT in individual human plasma samples. **g**, **h** NAD^+^ levels in recipient HEK293 cells (*n* = 3). 1% hP or hEV collected from a 5%-equivalent volume of plasma was added. **i**, **j** A scheme illustrating the eNAMPT-EVs administration protocol and NAD^+^ levels in the mouse hypothalamus at 30 min post-administration (*n* = 12). One, two, and three asterisks indicate *p* < 0.05, *p* < 0.01, and *p* < 0.001, respectively. Two-way ANOVA with Holm-Sidak’s multiple comparison test (**d**, **e**), one-way ANOVA with Holm-Sidak’s multiple comparison test (**g**, **h**) and unpaired Student’s t-test (**j**) were used. Data are presented as mean ± SEM.

eNAMPT-EVs are also contained in human plasma^13^. Thus, we examined whether human plasma-derived eNAMPT-EVs had the same NAD^+^ biosynthetic activity in HEK293 cells. Again, we were able to confirm the uptake of human eNAMPT in HEK293 cells (Fig. 2a, b) and detect significant increases in cellular NAD^+^ levels by adding human plasma (Fig. 2c, d). EVs purified from the same human plasma by ultracentrifugation, which eliminated a majority of albumin and transferrin relative to eNAMPT (Supplementary Fig. 2a), also significantly increased cellular NAD^+^ levels (Fig. 2e). To determine whether human eNAMPT was indeed responsible for these NAD increases, we used FK866, a potent NAMPT inhibitor. Even though FK866 moderately decreased NAD^+^ levels in control cells within 60 min (Supplementary Fig. 2b), the human plasma- or EV-dependent increases in cellular NAD^+^ levels were totally abrogated through pre-incubation of the plasma or purified EVs with FK866 (Fig. 2d and e), demonstrating that the elevation of NAD^+^ is due to human eNAMPT contained in EVs. Whereas most individual human plasma samples showed similar levels of circulating eNAMPT, two particular human plasma samples (hP-A and hP-B) had very high and very low levels of eNAMPT, compared to other plasma samples (Fig. 2f). Thus, we examined whether we were able to detect a difference in the NAD^+^ boosting effects of these two plasma samples in HEK293 cells. With a sufficient amount (10%) of each plasma sample added to the culture supernatant, we detected comparable increases in cellular NAD^+^ levels and were not able to distinguish these two plasma samples (Supplementary Fig. 2c). However, with the reduction to 1%, a significant difference in cellular NAD^+^ levels was detected between the two samples, although the dynamic range of detection was relatively limited (Fig. 2g). This difference was also detected upon the addition of EVs purified from each plasma (Fig. 2h). These increases in cellular NAD^+^ levels by hP-A and hP-B or their purified EVs were abrogated by pre-incubation with FK866 (Fig. 2g, h). To further examine whether ultracentrifuge-purified human eNAMPT-EVs can also boost NAD^+^ in vivo, EVs purified from combined human plasma samples were intravenously administered to naïve mice, resulting in significant increases in hypothalamic NAD^+^ levels (Fig. 2i, j). These results suggest that eNAMPT-EVs purified from human plasma can promote NAD^+^ biosynthesis in both cultured cells and mice.

### Exercise increases circulating eNAMPT and activates signaling pathways related to NAD^+^ metabolism in the hypothalamus

**Fig. 3:**
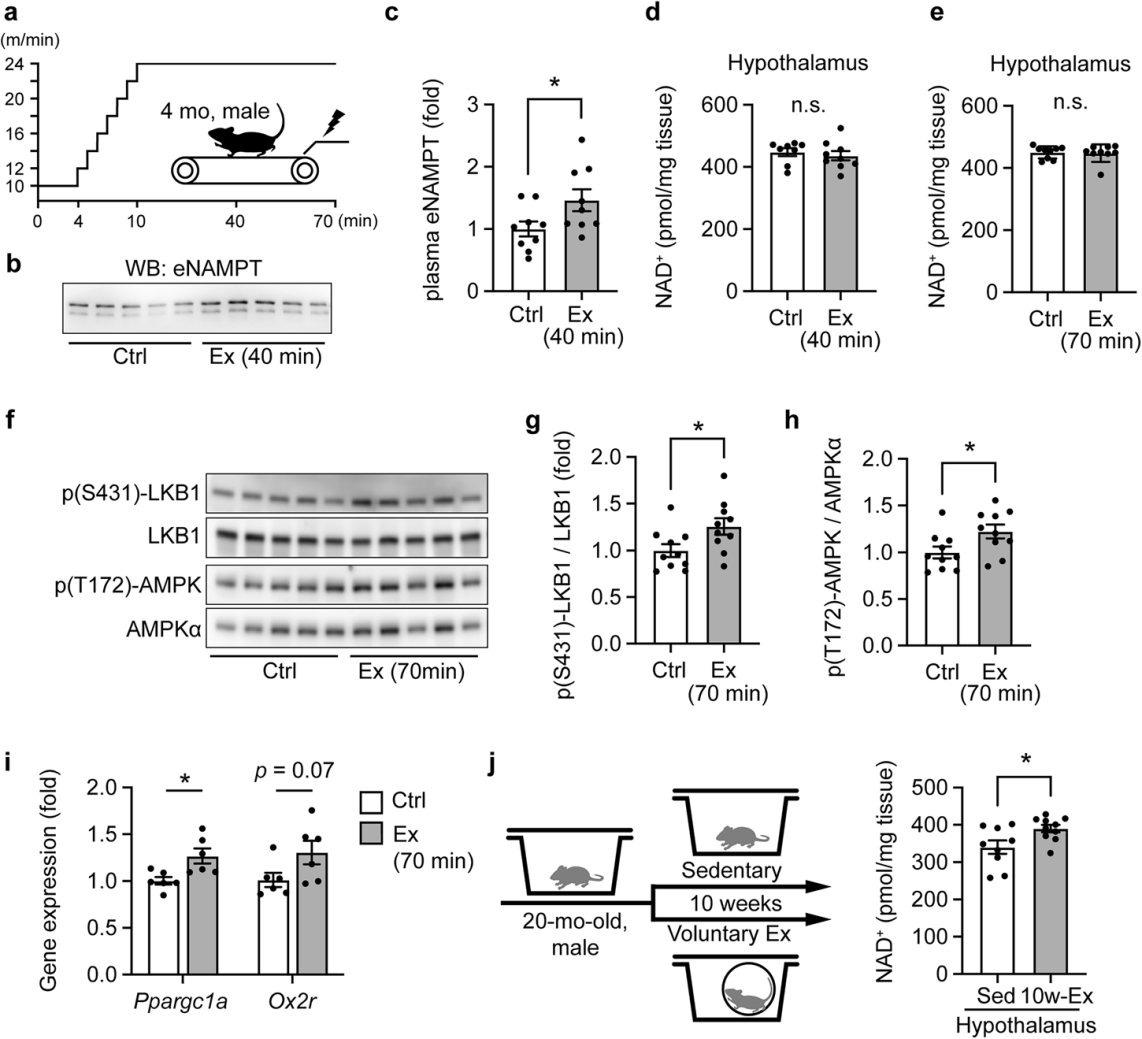
Exercise increases circulating eNAMPT and activates signaling pathways related to NAD^+^ metabolism in the hypothalamus. **a** A scheme showing the treadmill exercise protocol. Experiments were conducted at ZT12:00. **b**, **c** Representative immunoblot results of eNAMPT in mouse plasma following exercise (**b**), and the quantitated results of plasma eNAMPT levels before (Ctrl) and after exercise (Ex) (**c**) (*n* = 9). **d**, **e** Hypothalamic NAD^+^ levels after 40- and 70 min exercise. (**d**, *n* = 8–9; **e**, *n* = 9). **f**–**h** Representative immunoblot results and corresponding quantitated results of phosphorylated LKB1 and AMPK in the hypothalami 70 min after exercise initiation (*n* = 10). **i** mRNA expression levels of two SIRT1 target genes, *Ppargc1a* and *Ox2r*, in the hypothalamus 70 min after exercise initiation (*n* = 6). **j** The experimental protocol for voluntary exercise using aged mice (left) and hypothalamic NAD^+^ levels after 10 weeks (right) (*n* = 9–10). An asterisk indicates *p* < 0.05. Unpaired Student’s t-test was conducted to compare the results. Data are presented as mean ± SEM.

Exercise has been demonstrated to counteract aging in both mice and humans^20–23^. It has also been reported that a single bout of exercise increases circulating eNAMPT in young, physically active individuals^24^. Thus, we examined whether exercise could evoke increases in circulating eNAMPT and increase hypothalamic NAD^+^ levels in mice. When young mice were subjected to a 40 min treadmill exercise, including a 10 min acceleration phase, plasma eNAMPT levels were significantly increased in mice (Fig. 3a–c). Despite of these eNAMPT increases, no increases were observed in hypothalamic NAD^+^ levels after 40 min, and even after extending the sample collection time for additional 30 min (Fig. 3d, e). Such outcome could be expected if NAD^+^ turnover is also stimulated. If this is the case, we could detect readouts of increased SIRT1 activity in the hypothalami from exercised mice. Indeed, in the hypothalamus, we found that a single bout of exercise significantly enhanced phosphorylation levels of LKB1 and AMPK, two functional readouts of SIRT1 activity^25^ (Fig. 3f–h). Additionally, mRNA expression levels of *Ppargc1a* and *Ox2r*, two target genes of SIRT1^16,26^, showed significant increases or a trend of increase, respectively, in the hypothalamus (Fig. 3i). These results collectively suggest that NAD^+^ turnover may have been significantly increased in the hypothalamus after a single bout of exercise. Thus, we suspected that long-term exercise may induce detectable increases in steady-state NAD^+^ levels in the hypothalamus. Consistent with this idea, aged mice subjected to 10 week-long voluntary exercise indeed showed significant increases in hypothalamic NAD^+^ (Fig. 3j), suggesting that exercise is an important physiological stimulus to elevate circulating eNAMPT-EVs and enhance NAD^+^ levels in the hypothalamus, particularly when exercising for an extended period of time.

### Human-derived eNAMPT-EVs elicit physiological responses in mice

**Fig. 4:**
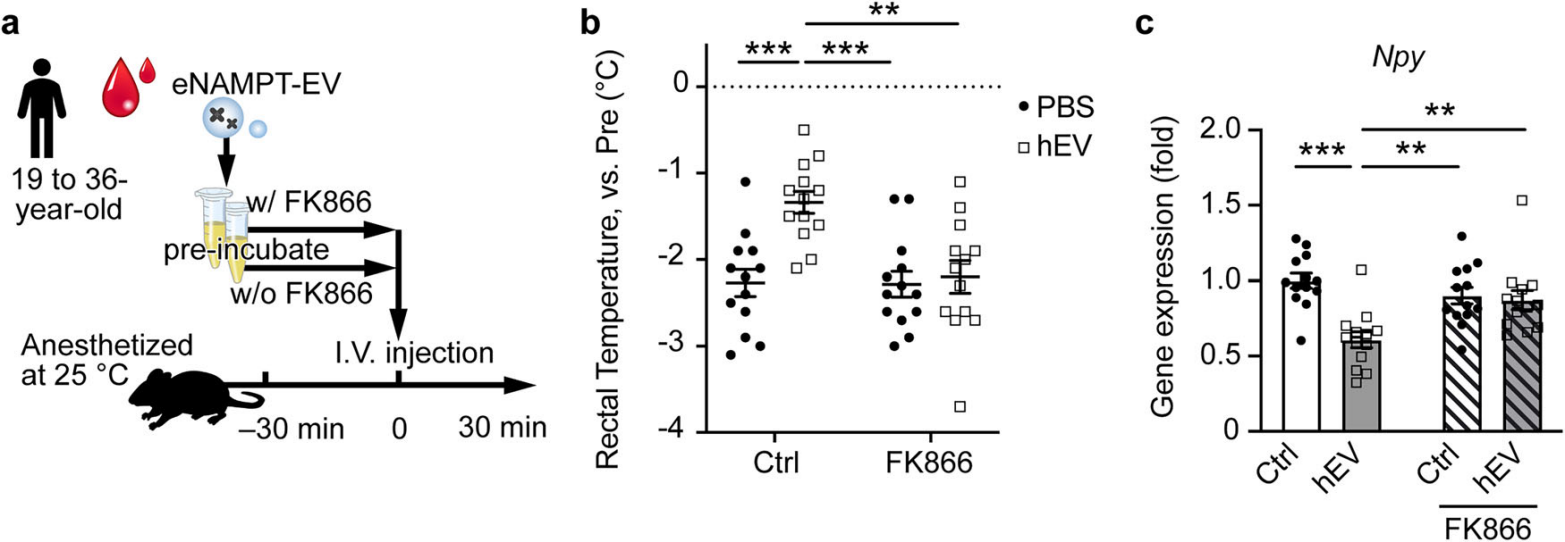
Human plasma-derived eNAMPT-EVs elicit physiological responses in mice. **a** A scheme illustrating the experimental protocol for human eNAMPT-EV administration experiments. Mice were kept under inhalation anesthesia without thermal support, with ambient temperature precisely maintained at 25 °C. FK866 was used at 0.1 mg per mouse (~5 mg/kg body weight) and pre-incubated with human plasma-derived EVs suspended in PBS. **b** Rectal body temperatures measured right before and 30 min after administration of human eNAMPT-EVs (hEV). Two-way ANOVA revealed a significant effect of hEV (*p* < 0.01) and FK866 (*p* < 0.01), and a significant hEV × FK866 interaction (*p* < 0.01) (*n* = 13). **c** mRNA expression levels of *Npy* in the hypothalamus 30 min post-administration of human eNAMPT-EVs. Two-way ANOVA revealed a significant effect of hEV (*p* < 0.001), no significant effect of FK866 (*p* = 0.14), and a significant hEV × FK866 interaction (*p* < 0.01) (*n* = 13). Two and three asterisks indicate *p* < 0.01 and *p* < 0.001, respectively. Two-way ANOVA with Holm-Sidak’s multiple comparison test was used (**b**, **c**). Data are presented as mean ± SEM.

Lastly, we investigated whether the administration of human plasma-derived eNAMPT-EVs could induce not only an increase in hypothalamic NAD^+^ but also physiological responses in mice. The hypothalamus is known as a central hub for the autonomic nervous system, regulating many fundamental body functions, including core body temperature. We found that under anesthesia, rectal body temperature was maintained at a significantly higher level 30 min after the intravenous administration of human eNAMPT-EVs. No elevation of inflammatory cytokines such as TNF-α or IL-6 was observed at 30 min time point following human eNAMPT-EV administration (Supplementary Fig. 3a). This effect on core body temperature was abolished by pre-incubation of human eNAMPT-EVs with FK866 (Fig. 4a, b), suggesting that this effect is due to human eNAMPT. It has been known that neuropeptide Y in the hypothalamus regulates core body temperature^27^, and knocking down *Npy* in the dorsomedial nucleus of the hypothalamus causes an increase in body temperature^28^. Consistently, mRNA expression levels of *Npy* in the hypothalamus were significantly suppressed in the human eNAMPT-EV-administered naïve mice, and this suppression was abolished by FK866 pre-incubation (Fig. 4c). Similar to *Npy*, mRNA expression levels of *Agrp*, which is involved in the regulation of energy metabolism in the hypothalamus^29^, exhibited similar changes in response to human eNAMPT-EVs alone and with FK866 (Supplementary Fig. 3b). Furthermore, in mice under an awake condition, an elevated body temperature was observed both at room temperature and following cold exposure after intravenous injection of purified human eNAMPT-EVs (Supplementary Fig. 3c), further providing compelling support for the NAD^+^ biosynthetic activity of human eNAMPT-EVs in mice. Taken together, human plasma-derived, highly purified eNAMPT-EVs not only induce an increase in hypothalamic NAD^+^ but also trigger functional physiological responses in mice.

## Discussion

In this study, we demonstrate that supplementing human plasma-derived eNAMPT-EVs can enhance NAD^+^ biosynthesis in the hypothalamus and induce physiological responses in mice. This NAD^+^ biosynthetic activity of purified human plasma-derived eNAMPT-EVs can be measured in the cell culture-based bioassay that we established in this study. Our results also emphasize the importance of exercise as a physiological stimulus to elevate circulating eNAMPT-EVs. Given that eNAMPT-EVs purified from young mice enhance hypothalamic NAD^+^ levels, ameliorate age-associated functional decline, and extend the maximum lifespan in aged mice^13,19^, these results strongly suggest the potential use of human eNAMPT-EVs as an effective anti-aging NAD^+^ booster.

Interestingly, the administration of purified human plasma-derived eNAMPT-EVs not only enhanced hypothalamic NAD^+^ levels in mice but also induced gene expression changes in the hypothalamus, leading to increased core body temperature. These findings strongly suggest that the function of eNAMPT-EVs is conserved between mice and humans. Most recently, it has been demonstrated that a specific subset of neurons in the dorsomedial hypothalamus (DMH), called DMH^Ppp1r17^ neurons, regulates white adipose tissue (WAT) function, including the secretion of eNAMPT-EVs, through the sympathetic nervous system, and plays a critical role in counteracting aging and extending lifespan in mice^18^. eNAMPT-EVs also enhance NAD^+^ biosynthesis in the DMH and the arcuate nucleus^19^, comprising a feedback loop between the hypothalamus and WAT. Given that circulating eNAMPT-EVs were enhanced by a single bout of exercise and that hypothalamic NAD^+^ levels were significantly increased by a long-term exercise, it is likely that the same feedback loop is involved not only in aging and longevity control but also in the physiological response to exercise. Although the relationship between exercise, increased circulating eNAMPT, and increased hypothalamic NAD^+^ still remains correlative and not causative, the observed link between exercise and eNAMPT may be important to understand how exactly exercise can help counteract the effects of aging.

It should be noted that all experiments in this study were performed using naïve mice that received a single administration of human plasma-derived EVs. It is also important to emphasize that no signs of acute immune activation were detected at 30 min time point when gene expression changes in the hypothalamus and corresponding alterations in core body temperature were observed, indicating that these effects are *not* due to an acute immune reaction. Nonetheless, because adaptive immune sensitization upon repeated EV injection could occur, we did not assess the effect of repeated EV injection in the present study. Therefore, in the near future, careful clinical studies will be needed to examine whether highly purified human plasma-derived EVs could work as an effective NAD^+^ booster and convey any health benefits in humans.

EVs contain many bioactive substances, including eNAMPT. For example, Wang et al. showed that EVs from the WAT of obese mice carry a microRNA that impairs cognitive function in mice^30^. In our case, the effects caused by the EVs purified from human plasma must be due to the NAD^+^ biosynthetic activity of eNAMPT contained in these EVs, because FK866, a potent NAMPT inhibitor, abolished the increase in cellular and tissue NAD^+^, gene expression changes in the hypothalamus, and their effect on core body temperature in mice. Although FK866 also inhibits intracellular NAMPT activity in recipient cells, we observed a significant statistical interaction between eNAMPT-EV administration and FK866 treatment, demonstrating that the observed effects were mediated by EV-derived eNAMPT. Because the content of EVs could be affected by the metabolic condition of WAT, it is crucial to carefully evaluate the quality of purified EVs which might reflect the condition of WAT and other tissues from which eNAMPT-EVs are released. In this regard, the bioassay system that we established in this study will be useful to conduct such an assessment, although the dynamic range of this bioassay needs to be further improved. The cell type used in this bioassay can also be customized, dependent on the effect of eNAMPT-EVs that investigators want to evaluate.

In this study, we relied on the measurement of cellular and tissue steady-state NAD^+^ levels to evaluate the effect of eNAMPT-EVs, due to technical limitations. This is most likely a significant reason why the dynamic range of our bioassay is relatively small. Whereas the measurement of steady-state NAD^+^ levels provides many valuable insights, it should be noted that such measurement allows only for a cross-sectional assessment of NAD^+^ metabolism, rather than a dynamic assessment of cellular and tissue NAD^+^ flux. To further elucidate the role and the impact of circulating eNAMPT-EVs in systemic NAD^+^ flux, further technical advancement will be necessary to accurately measure the NAD^+^ flux in vivo.

Whereas our findings in this study implicate the potential application of human eNAMPT-EVs as an effective anti-aging NAD^+^ booster or an exercise mimetic in humans, it should be aware that translating such encouraging outcome from mice to humans absolutely require careful, meticulous validation and technical development to ensure safety. Furthermore, in addition to the external administration of highly purified eNAMPT-EVs, strategies to enhance the release of eNAMPT-EVs from WAT will also be another interesting area of research in the near future. In conclusion, the present study reaffirms the intrinsic value of eNAMPT-EVs in developing an effective anti-aging intervention through systemic NAD^+^ boosting and thereby paves the way to achieve “productive aging”, with a cautious optimism for its pragmatic application in human clinical settings.

## Methods

### Cell culture

HEK293 cells were purchased from JCRB Cell Bank (JCRB9068). The HEK293 cells were maintained with DMEM (08458-45, Nacalai Tesque, Kyoto) supplemented with 10% fetal bovine serum (FBS) and 1% penicillin-streptomycin (168-23191, Wako, Osaka) at 37 °C with 5% CO_2_. OP9 cells were purchased from RIKEN BRC (RBC1124). The OP9 cells were maintained with αMEM (21445-95, Nacalai Tesque) supplemented with 20% fetal bovine serum (FBS) and 1% penicillin-streptomycin (168-23191, Wako, Osaka). Differentiation was induced by replacing the medium with αMEM supplemented with 15% Knockout Serum Replacement (10828028, Thermo Fisher Scientific) and 1% penicillin-streptomycin. The cells were cultured in this differentiation medium for four days.

### Preparation of *Nampt* knockdown adipocytes and their EVs

Knockdown of *Nampt* in OP9 cells was achieved using lentivirus, as previously described with minor modifications^16,31,32^. The *Nampt*-shRNA vector (target sequence: AGCGATAGCTATGACATTTAT) was designed and purchased from Vector Builder. *Luciferase*-targeting shRNA (target sequence: ATGTTTACTACACTCGGATAT) was used as a control. Undifferentiated OP9 cells at 50% confluency were prepared and infected with a mixture of lentiviral supernatant and growth medium containing 8 mg/mL polybrene at a 1:4 ratio for 24 h. Following infection, the OP9 cells were cultured in growth medium, passaged, and expanded until sufficient cell numbers were obtained. When the cells reached 100% confluency, the medium was replaced with a differentiation medium, and the cells were cultured for 4 days to induce differentiation into adipocytes with visible lipid droplets. At the start of differentiation induction, FBS was replaced with KnockOut Serum Replacement (ThermoFisher Scientific), a defined serum substitute not derived from animal serum, thereby reducing the risk of serum-derived EV contamination. For EV collection, the media were replaced with αMEM containing 1% penicillin-streptomycin, and the cells were incubated for 3 h. The collected supernatant was filtered using a 0.22 µm filter (Millex GP, Merck Millipore) pre-coated with fatty acid-free BSA in PBS. Ultracentrifugation was then performed at 32,000 rpm for 172 min using a Beckman SW 32 Ti rotor. The resulting pellet was resuspended in DMEM and used for experiments with HEK293 cells. Those EVs collected from OP9 adipocyte supernatants were concentrated approximately 500-fold.

### Mouse experiments

C57BL/6 J mice were purchased from the Jackson Laboratory Japan (Osaka). Mice were housed at 25 °C with 12 h light/12 h dark cycles and were fed a diet (CE-2, CLEA Japan, Tokyo) ad libitum with free access to water. Animal experiments and procedures were approved by the Institutional Animal Ethics Committees at the Institute of Biomedical Research and Innovation in the FBRI, Kobe, Japan (#20-02-02), the Institutional Animal Care and Use Committees of Kyoto Prefectural University, Kyoto, Japan (KPU260407), and the Animal Ethics Committee of National Center for Geriatrics and Gerontology, Obu, Japan (#5-50).

### Gene expression

RNA was extracted from frozen samples using ISOGEN II (Nippon Gene, Tokyo). RNA concentrations were determined using a NanoDrop spectrophotometer. cDNA synthesis was performed according to the product protocol of SuperScript IV VILO Master Mix (Thermo Fisher Scientific). For quantitative real-time PCR (qRT-PCR), either TaqMan Fast Advanced Master Mix or TB Green Premix Ex Taq II (Takara Bio, Shiga) was used. The qRT-PCR was conducted on a CFX Opus 96 Real-Time PCR System (Bio-Rad). *Glyceraldehyde 3-phosphate dehydrogenase* (*GAPDH*) or *TATA-box binding protein* (*TBP*) was used as internal standards for normalization. For detection, the following TaqMan probes and primers were utilized: *Ppargc1a* (Mm01208835_m1), *Ox2r* (Mm01179312_m1), and *GAPDH* (Mm99999915_g1). Primer sequences used were: *Npy* (Forward: GGACTGACCCTCGCTCTA, Reverse: TCGCAGAGCGGAGTAGTA), *GAPDH* (F: ACCCAGAAGACTGTGGATGG, R: GATGCAGGGATGATGTTCT), *TBP* (F: CAGCCTCAGTACAGCAATCAAC, R: TAGGGGTCATAGGAGTCATTGG), *Nampt* (F: AGATACTGTGGCGGGAATTG、R: GCTGCTGGAACAGAATAGCC) and *Agrp* (F: ACAACTGCAGACCGAGCA, R: GACGCGGAGAACGAGACT).

### Western blotting

Protein lysates were prepared from cultured cells or the hypothalamus with RIPA buffer (Nakalai Tesque) containing Halt protease inhibitor cocktail (Thermo Fisher Scientific) and phosphatase inhibitor cocktail (07574-61, Nakalai Tesque). SDS-PAGE was performed using Mini-PROTEAN TGX gels (Bio-Rad), and protein transfer was carried out utilizing the Trans-Blot Turbo Transfer System, following the manufacturer’s protocol. Primary antibodies were diluted in CanGetSignal solution A (Toyobo, Osaka) and incubated at 4 °C overnight. HRP-conjugated secondary antibodies diluted in CanGetSignal solution B (Toyobo) were incubated at room temperature for 1.5 h and visualized by chemiluminescence regents (ChemiLumi One Super, Nakalai Tesque) with a digital luminescent image analyzer Gel Doc XR (Bio-Rad). The signal intensities of protein bands were quantified using Fiji software (NIH). The following primary antibodies were used: NAMPT (A300-372A, Bethyl), Phospho(Ser428)-LKB1 (#3482), LKB1 (#3047), Phospho(Thr172)-AMPKα (#2535), AMPKα (#2532, Cell Signaling Technology), Transferrin (A80-128P, Bethyl), Albumin (16475-1-AP, Proteintech). In Fig. 2b and f, a custom-produced, rabbit polyclonal antibody against human NAMPT (Eurofins Genomics, Tokyo), which exhibits a strong reactivity to human plasma-derived eNAMPT, was used.

### NAD^+^ measurement

NAD^+^ quantification in cellular or tissue samples was performed using a high-performance liquid chromatography (HPLC) system (Shimadzu, Kyoto), equipped with a Supelco LC-18-T column (15 × 4.6 cm, Sigma) as previously described^19,33^. In brief, for tissue samples, tissue weights were accurately measured using an electronic balance equipped with an ionizer (AP225W-AD, Shimadzu) for normalization, whereas for cellular samples, cell counts from replicated wells with identical seeding densities were determined. Homogenization of samples was carried out in 10% perchloric acid, followed by a centrifugation at 20,000 × *g* for 5 min. The resultant supernatant was then neutralized using 3 M potassium carbonate (K2CO3), maintaining a 1:3 ratio with perchloric acid for 15 min, followed by another centrifugation at 20,000 × *g* for 5 min. The neutralized supernatant was subsequently mixed with a 5 M phosphate buffer at a 1:10 ratio and then transferred to an HPLC vial for analysis. The HPLC settings were as follows: A flow rate of 1 ml/min was maintained. The solvent system included 100% buffer A (50 mM phosphate buffer, pH 7.4) for the initial 5 min, transitioning through a linear gradient to 95% buffer A and 5% buffer B (100% methanol) over the next minute. This composition was kept constant from 6 to 11 min, followed by a gradient shift to 85% buffer A and 15% buffer B between 11 and 13 min. From 13 to 23 minutes, the system was operated with 85% buffer A and 15% buffer B, concluding with a gradient return to 100% buffer A in the final minute.

### EV isolation from plasma and injection

In the bioassay for eNAMPT-EVs, EVs were purified from both human and mouse plasma by ultracentrifugation. Human plasma was purchased from Biopredic International (PLA007). Human plasma was centrifuged at 2000 × *g* for 15 min and subsequently filtered through a 0.22 µm filter (Millex GP, Merck Millipore) that had been coated with fatty acid-free BSA in PBS. The filtered plasma was ultracentrifuged using a Beckman 70.1 Ti rotor at 50,000 rpm for 1 h. The resulting pellet was resuspended in PBS at 37 °C. FK866 (Adipogen) was used at a dose of 0.1 mg per mouse (approximately 5 mg/kg body weight) and was pre-incubated with the human plasma-derived EVs suspended in PBS for 3 hours. Mice were administered EVs derived from 20 mL of human plasma, which was a 1:1 male/female plasma mixture, via tail vein injection. All experiments using human plasma were conducted in accordance with the Declaration of Helsinki. For the injection of EVs from mouse plasma, the Total Exosome Isolation Kit from Plasma (ThermoFisher Scientific) was employed based on the manufacturer’s instructions. Mice were injected intravenously via the tail vein with EVs purified from 1.2 mL of mouse plasma collected on the same day. In all experiments, mice had no prior exposure to exogenous EVs, and EVs were administered only once.

### Rectal temperature measurement

Rectal temperature was measured using a mouse-specific probe (KN-91-AD1687, Natsume Seisakusho, Tokyo) under either inhalation anesthesia (Fig. 4b) or an awake state (Supplementary Fig. 2b). For the EV administration experiments under anesthesia, mice were maintained under 1.5% isoflurane inhalation anesthesia, and the ambient temperature was precisely monitored and kept at 25 °C. Thirty minutes after the initiation of anesthesia, rectal temperature measurement and EV administration were conducted, followed by another temperature measurement and sample collection after an additional 30 minutes under anesthesia. For the EV administration under the awake condition, 30 minutes post-injection, mice were transferred to a 4 °C cold room for 60-min cold exposure.

### Measurement of inflammatory cytokines

Ultracentrifuge-purified human plasma-derived EVs and LPS (Escherichia coli O55:B5; Sigma, L2637) at a dose of 1 mg/kg body weight were intravenously administered via the tail vein under anesthesia. Plasma samples were collected 30 min after injection, and TNF-α and IL-6 levels were measured using ELISA kits according to the manufacturers’ instructions (BMS607-3, Thermo Fisher Scientific; M6000B-1, R&D Systems).

### Treadmill exercise

Four-month-old male mice were subjected to treadmill exercise. Three days before the sampling, all mice were acclimated to a treadmill with an electric grid at the back (MK-690S, Muromachi Kikai, Tokyo). In the acclimation, the treadmill speed was set at 10 m/min and then increased by 3 m/min every minute, reaching a maximum speed of 25 m/min for 4 min. On the sampling day, treadmill exercise was started around ZT 12:00. For the first 4 min, the treadmill speed was set at 10 m/min. Then, the speed was accelerated by 2 m/min every minute until 24 m/min. At 40 min and 70 min (including 10 min of accelerating phase) or until the mice reached exhaustion, tissues were collected. Exhaustion was defined as the inability of the mouse to resume running after 5 s on the electric grid. Control mice were also brought into the lighted room where the treadmill exercise performed. Samples were collected from the control mice at the same time as the mice in the experimental group completed their exercise.

### Voluntary wheel-running

Male mice at 20 months of age were housed in voluntary running wheel cages (SN450, Sinano, Tokyo). Mice kept in these running cages ran ~4 km/day as an average. The day before sampling, during the light cycle, the mice were returned to their regular cages, and no wheel running was allowed during the final dark cycle. Samples were collected between ZT1:00 and ZT3:00 on the following day.

### Statistical analysis

Statistical analysis was performed in Prism 10 (ver. 10.1.1, GraphPad Software). For statistical comparisons of two conditions, a two-tailed Student’s t-test was used. For comparisons of more than two groups, the data were analyzed with one-way or two-way analysis of variance (ANOVA), according to the experimental design, followed by Holm-Sidak’s multiple comparison test. P values of *p* < 0.05 were considered statistically significant. All error bars show standard errors of means (SEM). n.s. indicates results that were not statistically significant.

## Supplementary information

Supplementary information.

## Data Availability

All data generated or analyzed during this study are included in this manuscript. Any additional materials are available from the corresponding author on reasonable request.
