# Supplementary material for: Human plasma-derived eNAMPT-containing extracellular vesicles promote NAD+ biosynthesis and thermogenesis in mice
Source: NPJ Aging. 2025 Nov 24;11(1):106. doi: 10.1038/s41514-025-00297-y (PMC12749881; doi:10.1038/s41514-025-00297-y)

Supplementary Information

Human Plasma-Derived eNAMPT-containing Extracellular Vesicles Promote NAD^+ Biosynthesis and Thermogenesis in Mice

Kiyoshi Yoshioka[1,2], Takumi Sugimoto[3], Mamoru Oyabu[3], Naoki Ito[2], Aoi Kodama[1], Yasutomi Kamei[3], Shin-ichiro Imai[1,4]

[1]Institute for Research on Productive Aging (IRPA), Tokyo, Japan.
[2]Brain-Skeletal Muscle Connection in Aging Project Team, Geroscience Research Center, National Center for Geriatrics and Gerontology, Obu, Japan
[3]Graduate School of Life and Environmental Sciences, Kyoto Prefectural University, Kyoto, Japan.
[4]Department of Developmental Biology, Department of Medicine, Washington University School of Medicine, St. Louis, MO, USA

*Correspondence:
Shin-ichiro Imai, M.D., Ph.D.
Theodore and Bertha Bryan Distinguished Professor
of Environmental Medicine
Department of Developmental Biology
Department of Medicine (Joint)
Washington University School of Medicine
MSC8103-0012-362
660 South Euclid Avenue
St. Louis, MO 63110
Tel: (314) 362-7228
Fax: (314) 362-7058
E-mail: imaishin@wustl.edu

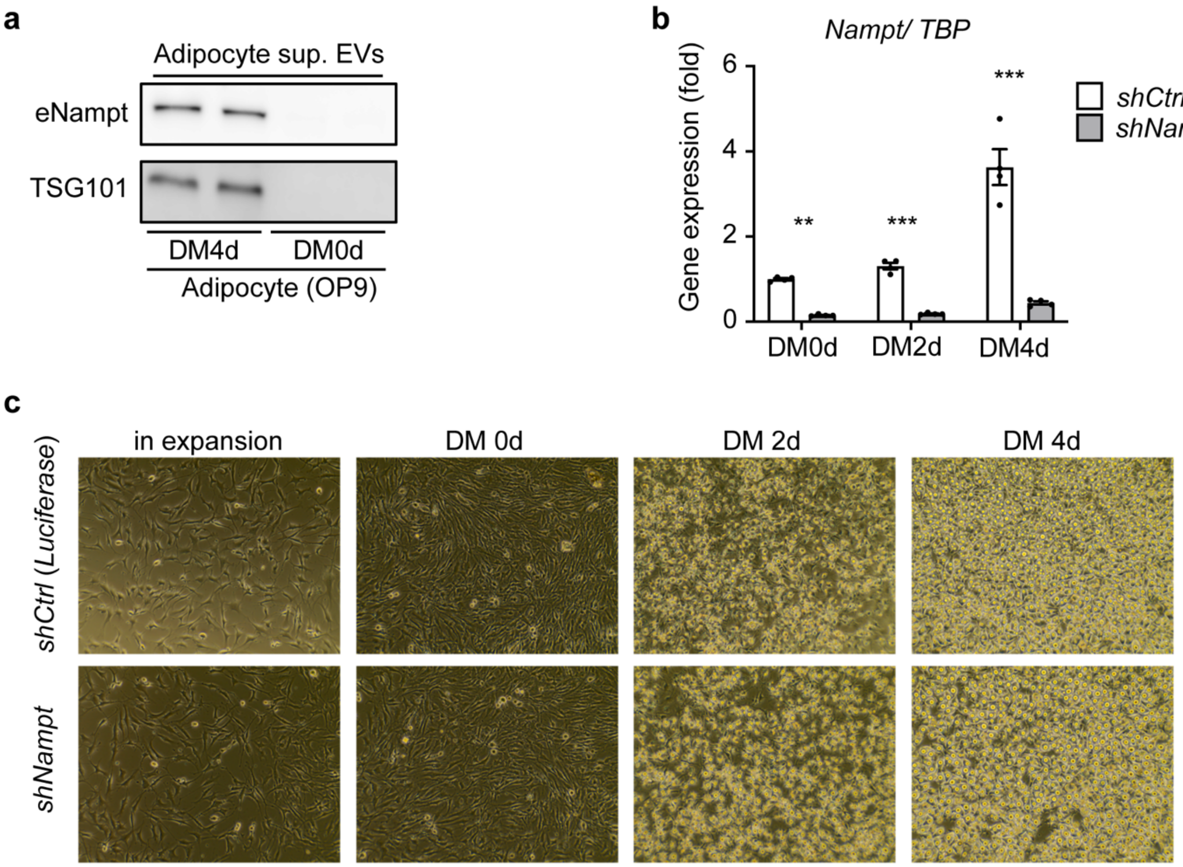

Supplementary Figure 1: Release of eNAMPT-EVs from cultured adipocytes and generation of *Nampt*-knockdown adipocytes.

(a) Western blot analysis of eNAMPT-EVs released into the culture supernatant by OP9 cells before and after differentiation into adipocytes (DM4d). Samples were prepared from EVs collected via ultracentrifugation from 2 mL of culture supernatant per lane. **(b)** RT-qPCR analysis of OP9 cells with *Nampt* knockdown induced by *shNampt* using lentivirus. Two-way ANOVA with Holm-Sidak's multiple comparison test were used and revealed a significant interaction between time and *Nampt* expression (time × *Nampt*/*TBP*; $p < 0.001$). (c) Phase-contrast images of *shCtrl* and *shNampt* OP9 cells after differentiation induction. Both cell types differentiated into adipocytes with lipid droplets after 4 days of induction. Data are presented as mean ± SEM. Two and three asterisks indicate $p < 0.01$, and $p < 0.001$, respectively.

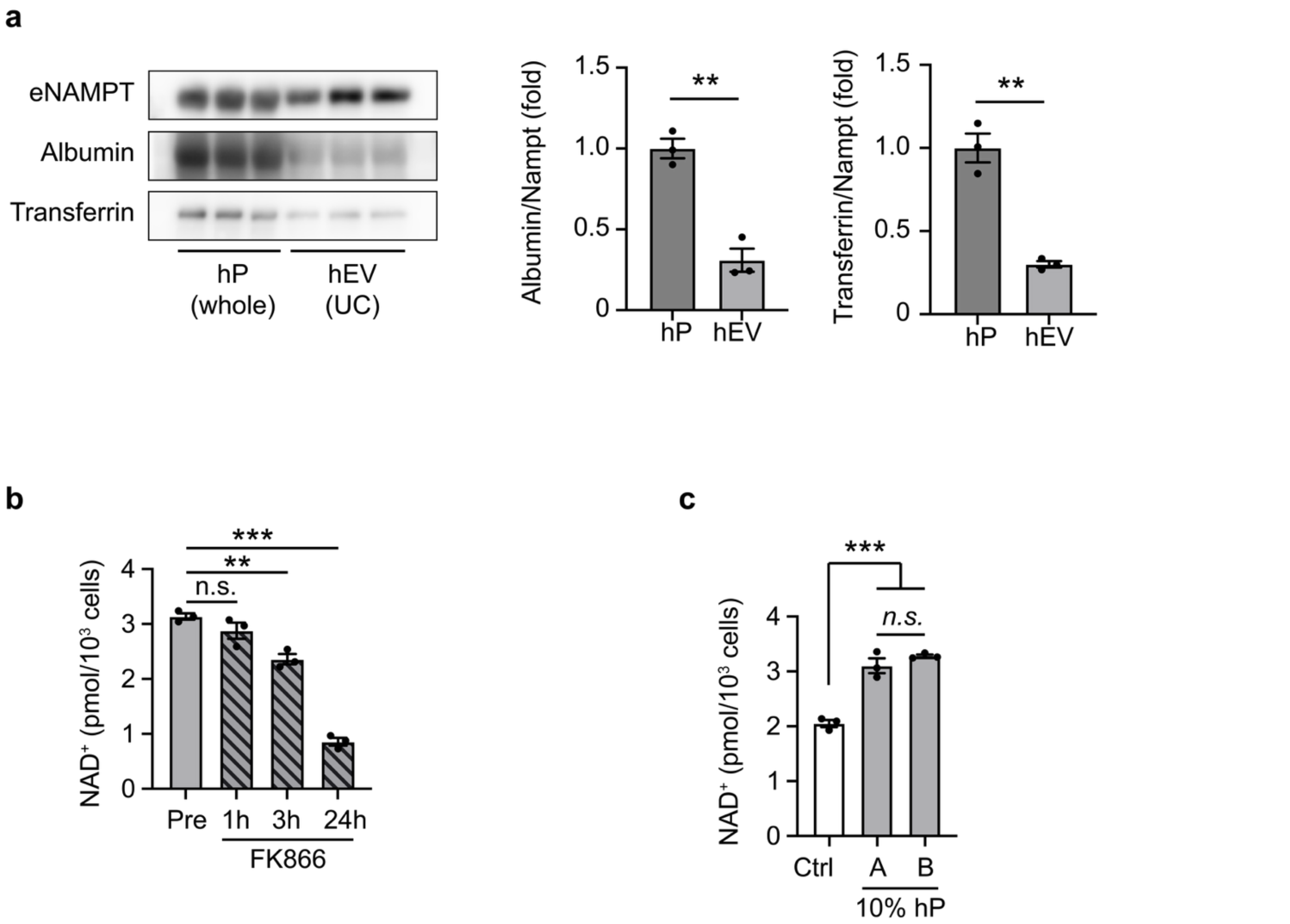

Supplementary Figure 2: Characterization of ultracentrifuge-purified human plasma-derived EVs and effects of a NAMPT inhibitor on iNAMPT and NAD$^+$ increase induced by the addition of 10% hP. **(a)** Western blot analysis of whole human plasma (hP) (0.1 μL/lane) and EVs purified from 2 μL of plasma by ultracentrifugation (hEV), with quantification of albumin and transferrin levels normalized to eNampt (n = 3). Statistical significance was determined by two-tailed unpaired Student's t-test. **(b)** Time course of NAD$^+$ changes upon addition of FK866 (100 nM) to the growth medium (10% FBS in DMEM). **(c)** NAD$^+$ levels in recipient HEK293 cells following the addition of 10% plasma sample from Fig. 2f. (n = 3). Cells treated with DMEM containing 1% penicillin but no FBS were used as a control (Ctrl). One-way ANOVA with Holm-Sidak's multiple comparison test were used (b, c). Data are presented as mean ± SEM. Two and three asterisks indicate $p < 0.01$, and $p < 0.001$, respectively.

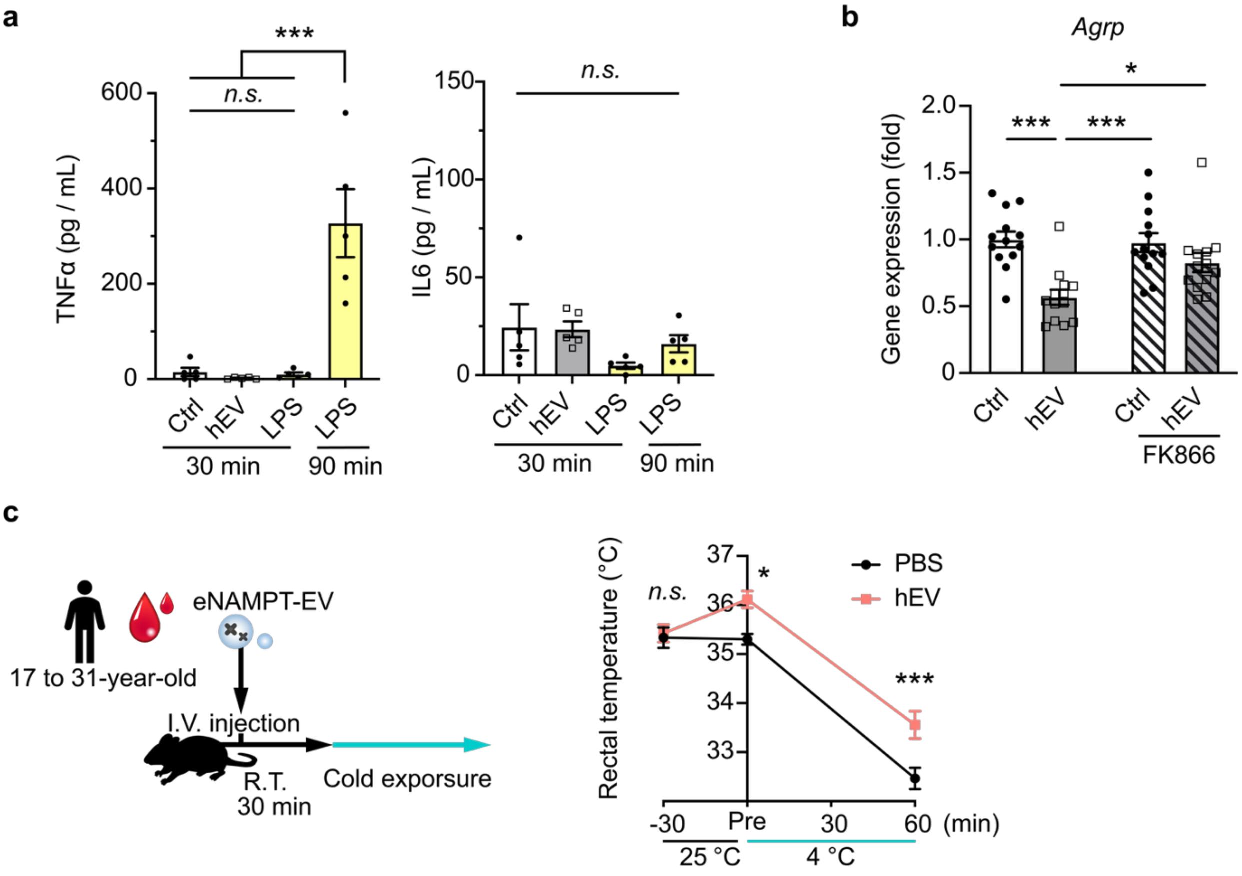

Supplementary Figure 3: The effects of ultracentrifuge-purified human eNAMPT-EVs on inflammatory cytokine levels, hypothalamic *Agrp* expression, and rectal temperature in mice..

(a) Plasma levels of inflammatory cytokines (TNF-α and IL-6) were measured 30 min after intravenous injection of ultracentrifugation-purified human eNAMPT-EVs (hEV), LPS (Escherichia coli O55:B5, 1 mg/kg), or PBS as a control. Plasma was collected, and cytokine levels were determined by ELISA ($n = 5$). **(b)** mRNA expression levels of *Agrp* in the hypothalami collected 30 min after eNAMPT-EV administration with or without pre-incubation with FK866. Two-way ANOVA revealed a significant effect of hEV ($p < 0.001$), no significant effect of FK866 ($p = 0.075$), and a significant hEV × FK866 interaction ($p < 0.05$) ($n = 13$). **(c)** The experimental protocol and quantitated results for rectal body temperature changes following human eNAMPT-EV administration. The experiments were conducted in non-anesthetized, awake conditions (n=14). One-way ANOVA with Holm-Sidak's multiple comparison test was used. Data are presented as mean ± SEM. One asterisk and three asterisks indicate $p < 0.05$ and $p < 0.001$, respectively.

Original uncropped and unprocessed Western blots

Figure 1a, NAMPT

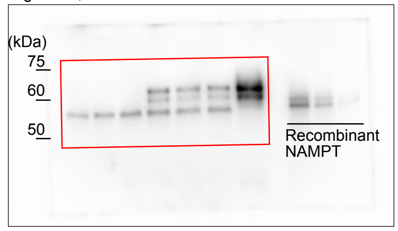

Figrue 1e, NAMPT

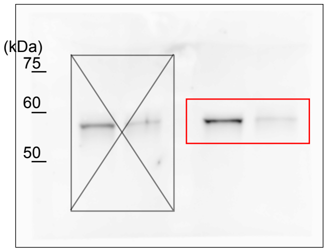

Figrue 1e, TSG101

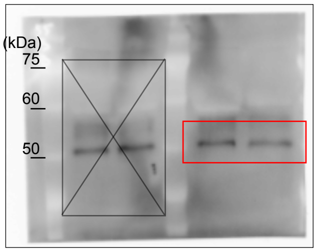

Figure 2b, NAMPT

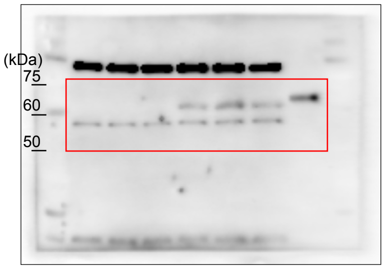

Figure 2f, NAMPT

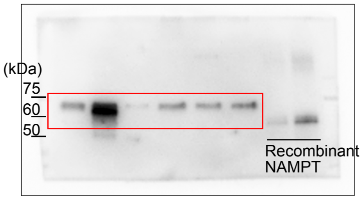

Figure 3f, p-AMPK

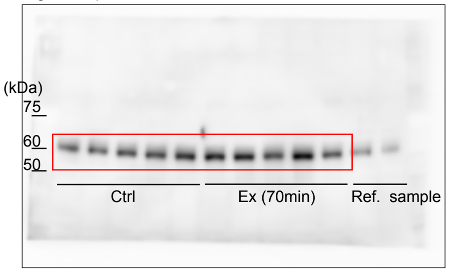

Figure 3b, NAMPT

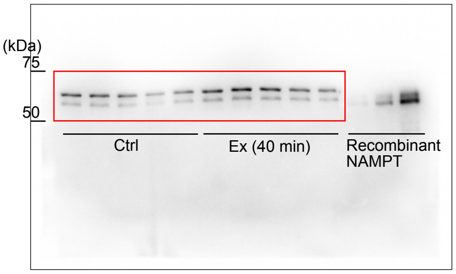

Figure 3f, p-LKB1

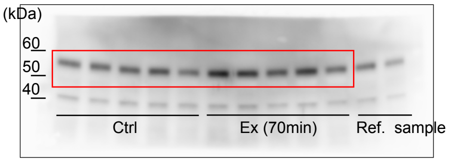

Figure 3f, LKB1

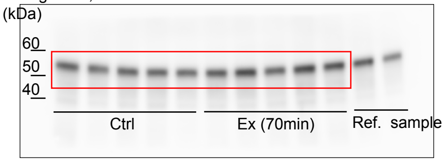

Figure 3f, AMPK

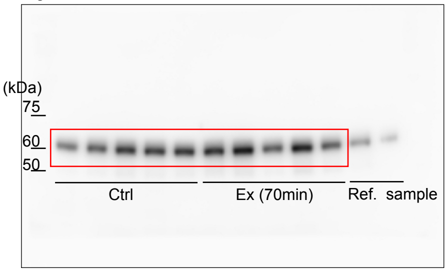

Supplementary Figure 1a, NAMPT

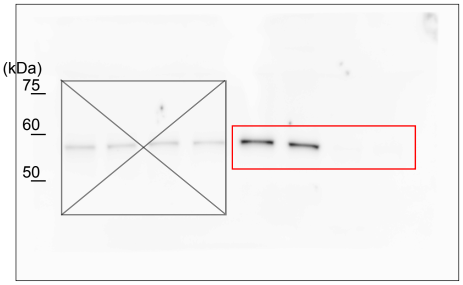

Supplementary Figure 1a, TSG101

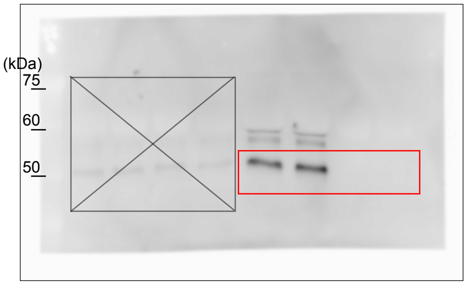

Supplementary Figure 2a, NAMPT

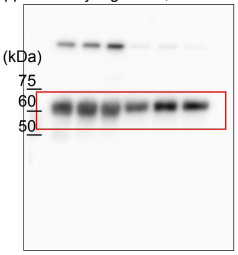

Supplementary Figure 2a, Albumin

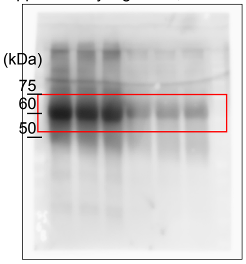

Supplementary Figure 2a, Transferrin

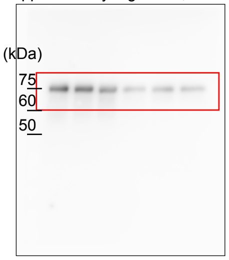

Supplement: Supplementary file 1 — Supplementary information. [file 41514_2025_297_MOESM1_ESM.pdf]
